# Different Etiologies of Dilated Pancreatic Duct Based on Endoscopic Ultrasonography Findings

**DOI:** 10.34172/mejdd.2024.382

**Published:** 2024-07-31

**Authors:** Elham Sobhrakhshankhah, Farhad Zamani, Hossein Ajdarkosh, Behdad Behnam, Amirhossein Faraji, Mahmoodreza Khoonsari, Mehdi Nikkhah, Ali Ajdarkosh, Fahimeh Safarnezhad Tameshkel, Dhayaneethie Perumal

**Affiliations:** ^1^Gastrointestinal and Liver Diseases Research Center, Iran University of Medical Sciences, Tehran, Iran; ^2^Commission for Academic Accreditation, Ministry of Education, Khalifa City, Abu Dhabi, UAE

**Keywords:** Pancreatic duct, Dilation, Neoplasm, Adenocarcinoma, Endoscopic ultrasonography

## Abstract

**Background:**

Pancreatic duct (PD) dilation could be presented in both benign and malignant diseases. Endoscopic ultrasonography (EUS) is a sensitive modality that provides both structural assessment and tissue sampling. This study aims to explore the importance of PD dilation as a potential indicator related to various pancreatobiliary pathologies identified via EUS.

**Methods:**

Among 3109 subjects who underwent EUS, 599 had evidence of dilated PD and met the inclusion criteria of this retrospective study. Also, the patients underwent EUS fine needle aspiration (EUS-FNA) to evaluate the etiology when required. All data were extracted from patients’ medical records to perform statistical analysis.

**Results:**

The study sample revealed 64% being male with a median age was 65-years. Pancreatic adenocarcinoma was the most common etiology diagnosed in 236 patients (39.4%), followed by sphincter of Oddi dysfunction (SOD) in 13% of subjects. Ampullary carcinoma, common bile duct stone, and cholangiocarcinoma were found at 9.5%, 8.8%, and 6.8%, respectively. Abdominal pain was the most common symptom seen in 440 (73.4%) patients. Opium consumption was reported in 170 (28.4%) subjects. Opium consumption was significantly more prevalent in patients with SOD (*P*<0.05).

**Conclusion:**

We suggest that PD dilation could be associated with a wide range of pancreaticobiliary pathologies, especially pancreatic neoplasms. In this regard, PD dilation should be considered as a crucial indicator of pancreatic neoplasm despite it may be associated with no clear etiologies.

## Introduction

 Pancreatic duct (PD) dilation could be defined as a mean diameter of more than 3 mm, 2 mm, and 1 mm in the pancreas’s head, body, and tail, respectively.^1^ It should also be noted that the PD could enlarge with age.^2^ In addition, PD dilation could also be found as an incidental abdominal imaging finding.^2^ The dilation’s etiology is diverse and could be presented in many conditions, including adenocarcinoma, pancreatic intraepithelial hyperplasia, neuroendocrine tumor, tumor metastasis, benign cyst, chronic pancreatitis, etc.^3^ However, it could be caused by pancreatic lesions (both benign and malignant) and is one of the major findings that lead to surgical resection of the pancreas.^4,5^ Therefore, it has been proposed that PD dilation needs more investigation.^6^ Numerous imaging modalities have been employed in order to evaluate PD abnormalities, including endoscopic ultrasonography (EUS), computed tomography (CT), magnetic resonance cholangiopancreatography (MRCP), and endoscopic retrograde cholangiopancreatography (ERCP).

 EUS is a relatively safe and sensitive technique for detecting pancreatic tumors.^7^ Furthermore, EUS provides a possibility of sampling from suspicious lesions using fine-needle aspiration (FNA) and fine-needle biopsy (FNB).^8^ This ability has improved malignant tissue detection, grading, and staging.^9^ Some studies have shown the cost-effectiveness of EUS and its power in tumor detection compared to other modalities, especially in those with pancreatobiliary duct dilation.^9^ There is limited data on different etiologies in those with PD dilation; nevertheless, we hypothesized that EUS/ EUS-FNA could be helpful in assessing the causes of PD dilation.^10^

 Given the scarcity of literature investigating the importance of PD dilation as a finding related to various pancreatobiliary pathologies, this study was implemented. In this regard, we evaluated different abnormalities in patients who had PD dilation on EUS.

## Materials and Methods

###  Study Design, Setting, and Participants

 This retrospective study was conducted from March 2020 to August 2022 at Firoozgar hospital affiliated to Iran university. The data of 3109 subjects referred for EUS for pancreatobiliary evaluation for any etiologies were examined. Of these subjects, 763 with PD dilation on EUS were selected based on the definition of PD dilation. No age or sex limitations were imposed to include patients. Further, 164 subjects were excluded if they had any of the following: (*a*) history of abdominal surgery that resulted in anatomical changes (like Roux-en-Y surgery), (*b*) incomplete data or duplicate patients, and (*c*) patients who underwent FNA when required, and experienced pathologists reported the cytology results. The study was conducted in concordance with the Declaration of Helsinki and its later revisions.

###  Variables, Data Sources, and Measurements

 The demographic data of included patients (age, sex, symptoms, opium/cigarette consumption status) were retrieved from their medical records. Three experienced gastroenterologists performed EUS/ EUS-FNA on patients who were monitored under general anesthesia. EUS was done using FUJIFILM processor (Tokyo, Japan) with linear endoscopes (EG-580UT, FUJIFILM, Tokyo, Japan), and aspiration was performed as indicated from visually suspected lesions using an echo-tip FNA needle (FG-32A, Cook Endoscopy, Winston-Salem, NC, USA). Using the EUS/EUS-FNA data, the final diagnosis was made. Extracted data from the selected patients’ medical records were used to perform statistical analysis.

###  Study Size 

 The following formula was used to calculate the sample size: (Z_1-α/2_)^2^(p)(1-p)/d^2^. Considering the population proportion of 0.06, type-one error of 0.05, and margin of error equal to 0.01, the calculated sample size was 216.^3^

###  Statistical Methods

 We used the SPSS software version 28 (IBM Corporation, N.Y., USA) to analyze the data. Qualitative variables were described as frequencies and percentages. Chi-squared test was used to compare the categorical data. Quantitative variables were reported as the median and interquartile range (IQR). The Shapiro-Wilk test was utilized to assess the continuous variables regarding normality. A *P* value of less than 0.05 was considered statistically significant.

## Results

###  Participants

 A total of 599 subjects with dilated PD, as detected via EUS, were evaluated regarding pancreatobiliary etiologies. 383 subjects were male (64%). The median age of the subjects was 65 years (IQR: 16); male subjects were significantly younger than female (median (IQR): 64-year-old (15) and 67-year-old (17), respectively; *P* = 0.003). 170 (28.38%) subjects had a history of opium consumption. Abdominal pain, jaundice, weight loss, pruritus, and fever were present in 440 (73.5%), 317 (52.9%), 313 (52.3%), 209 (34.9%), and 68 (11.4%) subjects, respectively.

###  Main Results

**Figure 1:**
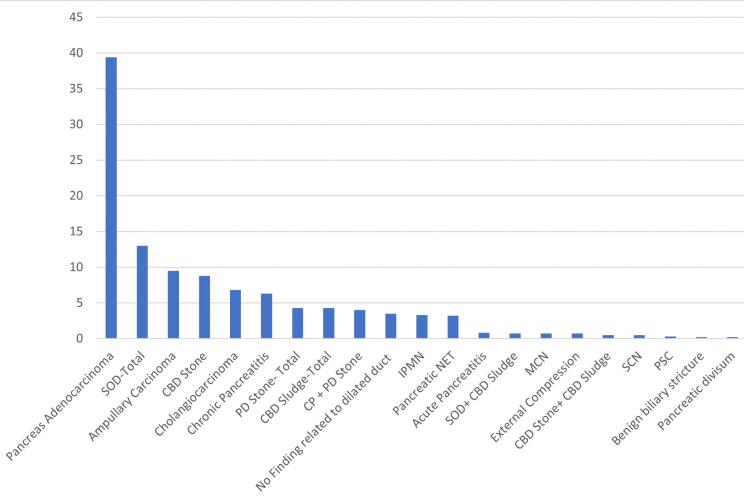
The frequency of pancreatobiliary pathologies in PD dilation

Figure 1 exhibits the etiologies of PD dilation as a bar chart. Pancreatic adenocarcinoma was diagnosed in 236 patients and was the most common finding in those with PD dilation (39.4%).

 Sphincter of Oddi dysfunction (SOD) was the second most prevalent cause seen in subjects (13%). Ampullary carcinoma, common bile duct (CBD) stone, cholangiocarcinoma, chronic pancreatitis, CBD sludge, and PD stone were in the next places (9.5%, 8.8%, 6.8%, 6.2%, 4.3%, and 4.3%, respectively). The frequency of other etiologies is shown in Table 1.

**Table 1:** Baseline characteristics of the participants

**Diagnosis**	**F** **(N)**	**T** **(N)**	**%**	**OC** **(Y)**	**AP** **(Y)**	**WL** **(Y)**	**JD** **(Y)**	**FV** **(Y)**	**PR** **(Y)**	**MA** **(year)**
Pancreatic adenocarcinoma	79	236	39.4	62	176	149	148	27	96	64.0
SOD-Total	32	78	13.0	41	54	33	34	6	21	63.0
Ampullary carcinoma	24	57	9.5	8	34	29	33	7	27	66.0
CBD stone	14	53	8.8	24	40	18	20	10	7	65.0
Cholangiocarcinoma	8	41	6.8	10	34	20	28	6	20	64.0
Chronic pancreatitis	12	38	6.3	10	33	19	13	2	8	65.0
PD Stone- Total	8	26	4.3	6	23	16	9	1	7	65.0
CBD sludge-Total	13	26	4.3	5	19	11	13	2	8	63.0
CP + PD Stone	10	24	4.0	5	22	14	9	1	6	63.0
No finding related to dilated duct	7	21	3.5	8	14	9	8	5	5	64.0
IPMN	7	20	3.3	3	15	9	5	1	2	70.0
Pancreatic NET	9	19	3.2	1	15	11	12	2	9	62.5
Acute pancreatitis	3	5	0.8	0	4	1	0	0	1	62.0
SOD + CBD sludge	2	4	0.7	0	2	2	3	2	2	66.0
MCN	4	4	0.7	0	3	2	2	0	3	70.0
External compression	1	4	0.7	0	3	1	4	1	2	49.0
CBD stone + CBD sludge	1	3	0.5	1	2	1	0	0	0	63.5
SCN	0	3	0.5	0	2	1	1	0	1	66.0
PSC	1	2	0.3	0	0	1	1	1	0	72.0
Benign biliary stricture	0	1	0.2	0	1	1	1	0	1	65.0
Pancreatic divisum	0	1	0.2	0	0	1	0	0	0	57

Legend: F: female; T: total; OC: opium consumption; AP: abdominal pain; WL: weight loss; JD: jaundice; FV: fever; PR: pruritis; MA: median age; N: number; Y: yes; SOD: sphincter of Oddi dysfunction; CBD: common bile duct; PD: pancreatic duct; CP: chronic pancreatitis; IPMN: intraductal papillary neoplasm; NET: neuroendocrine tumor; MCN: mucinous cystic neoplasm; SCN: serous cyst neoplasm; PSC: primary sclerosing cholangitis;

###  Other Outcomes

 We found no significant correlation between different dilated PD etiologies and sex or age (*P* = 0.51 and 0.28, respectively). We did not find any significant difference between the etiology groups regarding opium consumption except for those with SOD (*P* < 0.05). In this regard, 24.1% of patients with opium consumption had SOD on EUS, while only 8.6% of patients without a history of opium consumption had SOD (*P* < 0.05). Also, among symptoms, weight loss and jaundice were significantly higher in patients with pancreatic adenocarcinoma than in patients with other etiologies (weight loss: 63.1% vs. 45.1%; *P* < 0.05; jaundice: 62.7% vs. 46.5%; *P* < 0.05).

## Discussion

 In this retrospective study, we attempted to assess the prevalence of different disorders in those with PD dilation. We found that pancreatobiliary neoplasms are the most common etiology for PD dilation, which was seen in 63.4% of the subjects. Among them, pancreatic adenocarcinoma and ampullary adenocarcinoma were the most common type.

 Pancreatic cancer comprises 3% of all cancers in the US and has an annual incidence rate of 13.1 per 100 000 and an annual death rate of 11.1 per 100 000.^11^ This high mortality has been attributed to the difficulty in early diagnosis, which results in the presence of metastatic lesions at the time of diagnosis.^10^ A timely and accurate diagnosis could lead to better outcomes and decrease the mortality rate. Several modalities have been used to evaluate pancreas lesions, comprising CT, ERCP, MRCP, and EUS.^9,10^ EUS is the preferred approach to assess suspicious lesions in the pancreas. FNA could be performed in case of any pathological finding on EUS when tissue sampling evaluation is indicated. These advantages make it possible to diagnose malignancies effectively and determine the tumor’s grading and staging.^10^

 As mentioned earlier, pancreatic cancers might have no symptoms, making the prompt diagnosis challenging. In some cases, incidental findings could be helpful as they could be caused by malignant and pre-malignant lesions.^2^ These incidental findings are expected to grow due to the increased number of abdominal imaging.^12^ Therefore, using these data correctly could lead to the early diagnosis of malignancies. Given the high burden of pancreatic lesions, these abnormalities must be carefully investigated.^2^ Still, there is limited evidence of underlying etiologies of PD dilation.

 In a retrospective study performed by Agarwal and colleagues, the prevalence of pancreatic neoplasms was assessed in 110 subjects, with imaging results suggestive of a dilated PD or enlarged pancreatic head using EUS/EUS-FNA.^3^ They found that seven patients had adenocarcinoma, one had pancreatic intraepithelial neoplasia, one suffered from neuroendocrine tumor, one was afflicted with tumor metastasis, and three had benign cysts. Chronic pancreatitis was present in 32 cases, and no pathological finding was seen in the other 65 subjects. In those with dilated PD plus a sudden cutoff, adenocarcinoma was seen in 33.3% of cases. They also proposed that EUS-FNA is a helpful technique for diagnosing pancreatic neoplasms with an accuracy of 99.1%, a sensitivity of 88.8%, a specificity of 100%, a negative predictive value of 99%, and a positive predictive value of 100%. Their study was limited by a small number of patients; still, they concluded that EUS-FNA should be done in those with an enlarged pancreatic head and those with dilated PD, given the relatively high prevalence of neoplasm.

 Carriere and colleagues conducted a retrospective database analysis in a tertiary hospital on patients with dilated CBD with/without PD dilation who had no recognized etiology.^12^ They assessed the data of 140 patients and found that only 18 patients (12.9%) had a malignant or pre-malignant lesion [intraductal papillary mucinous neoplasm (5%), pancreatic adenocarcinoma (4.3%), ampullary tumor (2.14%), and mucinous cystic neoplasm (1.4%)]. They reported that 36 patients (25.7%) had benign disorders [including choledolithiasis (7.9%) and chronic pancreatitis (6.4%)]; however, the majority of subjects (61.4%) had dilated ducts without specific etiology. They also suggested that EUS could be used in those unexplained duct dilations to assess the underlying cause.

 In another study performed by Roberts and colleagues, 61 patients with dilated PD with or without dilated CBD were retrospectively investigated.^13^ The etiology was not identified in 38 subjects; five patients had pancreas cancer, four had intraductal papillary mucinous neoplasm, two had a suspicious ampullary tumor, one had cholangiocarcinoma, and one had mucinous cystadenoma. The remaining patients had benign conditions, including biliary stone disease, chronic pancreatitis, pseudocyst, choledochal cyst, and pancreas divisum.

 Gupta and co-workers reported that pancreatic lesions in the absence of PD dilation are suggestive of neuroendocrine tumors (NETs) (the dilation was seen only in 3.3%), while PD dilation is more likely to be observed in cases with pancreatic adenocarcinoma (88.9% of cases with primary adenocarcinoma).^1^

 In a study on 524 Japanese subjects with PD dilation, pancreatic diseases were seen in 24.8% of the study population (pancreatic cysts: 15.6%, chronic pancreatitis:4.9%, and malignancies: 1.3%).^14^

 In line with previous studies, our results showed that opium consumption was associated with SOD due to its effect on increasing sphincter Oddi tonicity.^15,16^ Narcotic agents have known as an effective, responsible factor for SOD.^16,17^ Studies have also suggested chronic use of opiates and its derivatives may lead to biliary stasis and CBD dilation.^18,19^ Although the effect of opium on SOD is complex and multifactorial, Wu and colleagues^16^ showed that morphine consumption led to an increase in both the amplitude and frequency of phasic contractions of Oddi’s sphincter.

 As mentioned earlier, our result showed that the prevalence of neoplasms, especially pancreatic adenocarcinoma, in these patients is considerably higher than in the previous studies. This could be because this study was performed in a single tertiary center with advanced diagnostic tools. However, further evaluation with multi-center studies is needed before conclusions can be reached.

## Strengths and Limitations

 This study was empowered with a relatively big sample size. However, it encountered some drawbacks, first: limited variables did not let us investigate possible correlations of different factors with dilated PD etiologies. Second, we could not access patients’ other data, and consequently, we could not assess confounding factors. Third, our study was done in the setting of a tertiary center, which results may be different from the normal population.

## Conclusion

 This study showed a high prevalence of pancreatic neoplasms in subjects with dilation of PD. We suggest that dilated PD is associated with a broad spectrum of pancreatobiliary pathologies from benign to malignant, even without clear etiology, and should be carefully investigated.
